# Questionnaire Survey to Identify the Medical Departments That Patients With Possible Head and Neck Cancer (HNC) Symptoms Tend to Choose

**DOI:** 10.7759/cureus.55715

**Published:** 2024-03-07

**Authors:** Kohei Matsumoto, Fujinobu Tanaka, Yoshihiko Kumai

**Affiliations:** 1 Department of Otolaryngology-Head and Neck Surgery, Nagasaki University Graduate School of Biomedical Sciences, Nagasaki, JPN; 2 Department of Otolaryngology-Head and Neck Surgery, National Hospital Organization (NHO) Nagasaki Medical Center, Omura, JPN; 3 Department of Otolaryngology-Head and Neck Surgery, Nagasaki University Graduate School of Medicine, Nagasaki, JPN

**Keywords:** questionnaire survey, delay in treatment initiation, neck masses, symptoms, hnc, head and neck cancer

## Abstract

In the treatment of head and neck cancer (HNC), any delay in omit initiation worsens the overall prognosis. Thus, the early start of HNC treatment is crucial. Unfortunately, treatment delays persist in clinical practice. There are several possible reasons for this. One reason is that patients with HNC do not visit an ear, nose, and throat (ENT) doctor. This is because non-ENT doctors (e.g., general practitioners {GPs}) lack expertise in HNC and therefore may unrecognize it. Therefore, guiding patients with suspected HNC symptoms to an otorhinolaryngologist, an HNC specialist, is necessary. To determine the departments that patients with potential HNC symptoms tend to select, we administered a questionnaire survey to 140 participants. Fewer than 60% of respondents indicated they would consult an otorhinolaryngologist even when recognizing symptoms suggestive of HNC. Notably, a significantly low percentage of respondents mentioned they would consult an otorhinolaryngologist for neck masses. Public awareness of HNC symptoms, especially the association between a neck mass and HNC, is limited. The lack of understanding by the general public regarding the relationship between neck masses and HNC is a challenge to prompt initiation of treatment.

## Introduction

Several reports regarding the treatment of head and neck cancer (HNC) have indicated that any delay in the treatment initiation can affect the prognosis [1-4]. Consequently, it is crucial to ensure an immediate start to chemoradiotherapy for HNC. Nevertheless, clinical practice often witnesses delays in treatment initiation for various reasons. Multiple factors contribute to these delays, and one notable factor is the initial consultation with a general practitioner (GP). Even if a patient has seen a GP, an early diagnosis can be made by scheduling a follow-up visit. However, if GPs are unable to recognize HNC at the first visit and a follow-up visit cannot be scheduled, treatment may be delayed. [5]. GPs who lack expertise in HNC are more prone to misdiagnosis [6,7] and may unrecognize HNC cases [8]. Thus, we believe it is essential to guide patients with symptoms suggestive of HNC to consult an ear, nose, and throat (ENT). To identify the departments that patients with potential HNC symptoms tend to select, we conducted a questionnaire survey.

## Materials and methods

An awareness survey was conducted on symptoms specific to HNC among first-time patients visiting the Department of Otorhinolaryngology at the National Hospital Organization Nagasaki Medical Center between February 1, 2021, and March 31, 2022. To ensure the inclusion of patients with no prior knowledge of HNC, we selected patients who sought consultation for conditions, such as otitis media, hearing loss, dizziness, facial paralysis, or sinusitis. This research theme was identified in advance before the survey. The questions were created by the author, and the survey began after a pilot test. There was no interviewer; the clerk distributed questionnaires while waiting for the consultation, and the patients were asked to voluntarily respond. When distributing the questionnaire to the participants, we obtained consent for study participation. Administrative staff and nurses assisted subjects when necessary. In addition, we explained to the clerks and nurses in advance that this was a cancer awareness survey. Repeat interviews were not conducted. No audio or video recordings were used. Field notes were created after all questionnaire surveys were completed. There was no exchange of opinions or discussion about data saturation during the survey implementation period. Transcripts were not returned. The authors coded the data obtained. Statistical analysis was performed using JMP Pro 14.2.0 (SAS Institute Inc., Cary, NC). Based on the responses that were collected, Fisher’s exact test was employed to compare the proportion of patients who initially chose ENT for HNC-specific symptoms with those who chose non-ENT. Moreover, Fisher’s exact test was used to compare the number of patients in the early and non-early presentation group.

**Figure 1:**
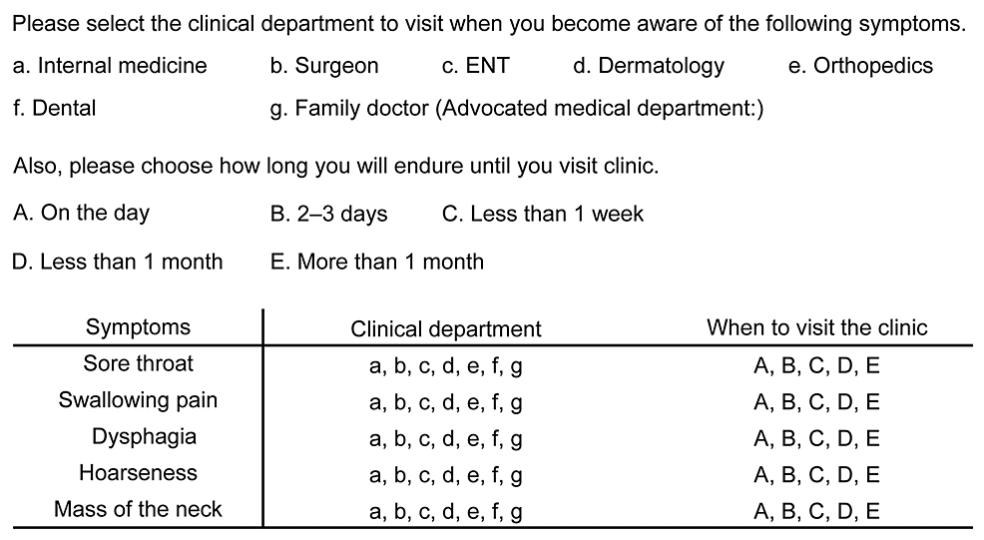
Content of the questionnaire survey. This indicates the content of the questionnaire survey. This questionnaire was created by the author. New patients eligible for the survey were asked to fill out a note while waiting for their consultation.

Figure 1 shows the content of the questionnaire. First, we assessed the awareness of HNC-related symptoms, including sore throat, pain when swallowing, dysphagia, hoarseness, and neck mass. In particular, patients were presented with a questionnaire asking them to select from a list of potential medical specialties, including internal medicine, surgery, ENT, dermatology, orthopedics, dentistry, or their family doctor as their preferred place for consultation. Based on the collected responses, the departments visited for each symptom were divided into ENT and non-ENT groups and analyzed. Patients who chose a family doctor, which is unique in Japan, were asked to specify the medical department represented by their family doctor. Those who did not provide a department choice were excluded from the analysis for that symptom. 

In addition, we investigated the period from the time when patients noticed symptoms specific to HNC until they visited a medical institution. In particular, we conducted a questionnaire survey in which participants were asked when they would consider visiting a doctor: on the day they noticed symptoms, after two to three days, within one week, within one month, or over one month. Symptoms for which we could not obtain information regarding when to visit a doctor were excluded from the analysis. Based on the responses we received, individuals who opted to seek medical treatment on the day they noticed symptoms, within two to three days, or within seven days were categorized as the “early consultation group.” Those who chose to seek medical attention within one month or more than one month after symptom onset were classified as the “non-early consultation group.” The rationale for setting the cutoff at seven days and grouping patients into early and non-early examination groups is explained below. It was reported that the tumor volume of HNC increases in 70% of cases within 28 days [9], so treatment should be started as soon as possible after the onset of symptoms before the occurrence of adverse effects, such as tumor growth. It was reported that the risk of death increases when the period from pathological diagnosis to the start of treatment exceeds 46-52 days [1]. Meanwhile, the period from pathological diagnosis to treatment initiation was reported to be 34 days [10], 35 days [11], 37 days [4], and 48 days [12]. Although there are differences between reports, considering the time required to prepare for treatment, we believe that in order to receive the maximum benefit of improving the prognosis through treatment, it is desirable to see a doctor approximately one week after symptom onset. Therefore, the cutoff value was set at seven days, and the patients were classified into an early consultation group and a non-early consultation group. For all analyses, p < 0.05 was considered statistically significant. This study was approved by the Ethics Committee of the National Hospital Organization Nagasaki Medical Center (registration number: 2020124). The authors attended a training session on clinical research conducted by the Nagasaki University Hospital Clinical Research Center. In addition, the authors took the research ethics e-learning material (eAPRIN) that is compulsory at the National Hospital Organization Nagasaki Medical Center.

## Results

Consent was obtained in all cases, and there were no nonparticipants. Table 1 shows the basic demographic characteristics of participants. The target audience was 140 people. Of these, 15 were only able to provide a partial answer to the question about the medical department they were visiting. The number of non-respondents by symptom was six for sore throat, six for pain when swallowing, seven for dysphagia, 11 for hoarseness, and 11 for neck mass. Forty-two people selected their primary care physician in one of the questionnaires, but all of the departments they advocated were internal medicine, so cases that selected internal medicine and family medicine were treated as the internal medicine group. Additionally, 20 people received only partial answers to questions about when they should seek medical attention. The number of non-respondents by symptom was nine for sore throat, eight for pain when swallowing, eight for dysphagia, 15 for hoarseness, and 11 for neck mass. 

**Table 1:** Basic demographic characteristics of participants (N = 140).

Variables	Category	N (%)
Gender	Male	62 (44.3%)
	Female	78 (55.7%)
Age group	20-29 years	20 (14.3%)
	30-39 years	32 (22.9%)
	40-49 years	32 (22.9%)
	50-59 years	13 (9.3%)
	60-69 years	24 (17.1%)
	70-79 years	15 (10.7%)
	>79 years	4 (2.9%)

**Figure 2:**
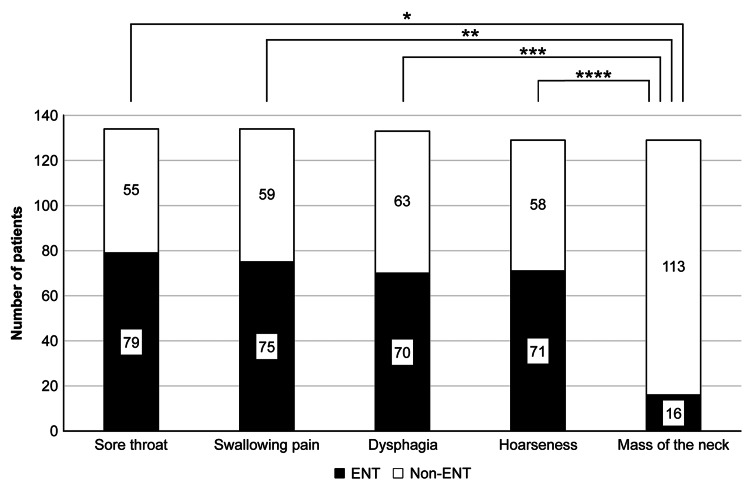
Number of patients in the ENT group and non-ENT group for each symptom. Based on the answers obtained, patients were classified into those who chose ENT and those who chose non-ENT for HNC-specific symptoms. We compared the proportion of patients choosing ENT for neck masses and those choosing ENT for other symptoms. *p < 0.001, **p < 0.001, ***p < 0.001, **** p < 0.001, according to Fisher’s exact test.

Table 2 shows the number of departments visited for each symptom, and Figure 2 shows the number of patients in the ENT group and non-ENT group for each symptom. In all, 79/134 people (59.0%) answered that they would consult an ENT for sore throat, while 75/134 people (56.0%) answered that they would consult an ENT for pain when swallowing. For dysphagia, 70/133 people (52.6%) consulted an ENT. For hoarseness, 71/129 people (55.0%) consulted an ENT. However, only 16/129 people (12.4%) mentioned that they would consult an ENT for a neck mass. Less than 60% of respondents answered that they would consult an ENT for any symptoms, and the number of respondents who answered that they would consult an ENT for a neck mass was particularly low. This was significantly lower than the proportion of respondents who said that they visited an ENT for sore throat, odynophagia, dysphagia, and hoarseness, all with p-values <0.01 based on Fisher’s exact test. The department most frequently chosen for the treatment of neck masses was the internal medicine group, with 42 patients (33.6%). Of these, 28 were in internal medicine, and 24 were in internal medicine as family physicians. The next most common field was dermatology, with 36 people (25.7%).

**Table 2:** First department selected according to the symptoms. This indicates the medical department selected for each symptom.

	ENT	Non-ENT					
Symptoms		Internal medicine	Family doctor	Surgeon	Orthopedics	Dermatology	Dental
Sore throat	79	55					
	59.0%	24 (17.9%)	27 (20.1%)	3 (2.2%)	0	0	1 (0.7%)
Swallowing pain	75	59					
	56.0%	27 (20.1%)	25 (18.7%)	3 (2.2%)	0	0	4 (3%)
Dysphagia	70	63					
	52.6%	33 (24.8%)	22 (16.5%)	1 (0.8%)	0	0	7 (5.3%)
Hoarseness	71	58					
	55.0%	27 (20.9%)	26 (20.2%)	2 (1.6%)	0	1 (0.8%)	2 (1.6%)
Mass of the neck	16	113					
	12.4%	19 (14.7%)	24 (18.6%)	21 (16.3%)	11 (8.5%)	38 (29.5%)	0

**Figure 3:**
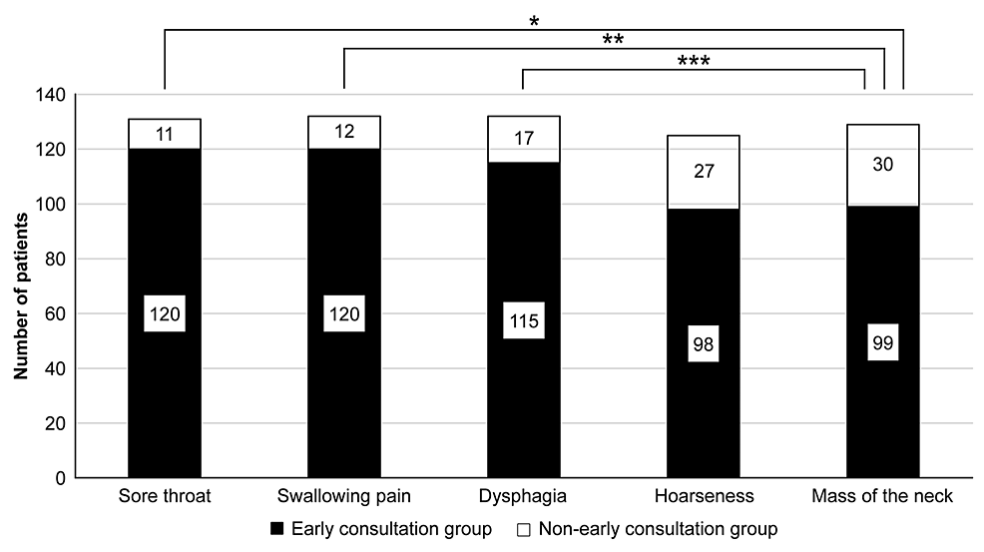
Number of patients in the early diagnosis group and non-early diagnosis group for each symptom. Based on the answers obtained, patients were classified into those who seek early medical care for head and neck cancer (HNC)-specific symptoms and those who seek non-early medical care. We compared the proportion of patients choosing early consultation for neck masses and those choosing early consultation for other symptoms. *p = 0.001, **p = 0.002, ***p = 0.036, according to Fisher’s exact test.

Table 3 shows the estimated number of days it would take patients to see a doctor if they noticed symptoms. Figure 3 shows the number of patients in the early diagnosis group and non-early diagnosis group for each symptom. The proportion of patients in the early consultation group for sore throat was 120/131 people (91.6%). The rate of pain during swallowing in the early consultation group was 120/132 people (90.9%). The proportion of dysphagia in the early consultation group was 115/132 people (87.1%). The proportion of patients with hoarseness in the early consultation group was 98/125 people (78.4%). The proportion of patients with neck masses in the early consultation group was 99/129 people (76.7%). The proportion of patients with neck masses in the early diagnosis group was significantly lower than that with sore throat, pain on swallowing, and dysphagia, with p values of 0.001, 0.002, and 0.036, respectively (Fisher’s exact test).

**Table 3:** Period between the symptom onset and the visit to a medical institution. This indicates the selected timing for consultation for each symptom.

	Early consultation group			Nonearly consultation group	
	On the day	2–3 days	<1 week	<1 month	>1 month
Sore throat	120 (91.6%)			11 (8.8%)	
	10	62	48	10	1
Swallowing pain	120 (90.9%)			12 (9.1%)	
	10	60	50	10	2
Dysphagia	115 (87.1%)			17 (22.9%)	
	12	55	48	15	2
Hoarseness	98 (78.4%)			27 (21.6%)	
	6	38	54	20	7
Mass of the neck	99 (76.7%)			30 (23.3%)	
	19	28	52	21	9

## Discussion

Our questionnaire survey revealed that nearly 40% of patients opt for internal medicine as their medical department for the treatment of HNC-specific symptoms. Despite Japan’s medical system allowing patients to freely choose their attending department, there remains a possibility that a significant portion of HNC patients may select a department other than otolaryngology. In particular, patients are inclined to choose internal medicine or a family medicine clinic where family doctors provide health consultations. Similar reports exist, although making a direct comparison can be challenging due to differences in the medical system in Japan. Notably, in some cases, patients visit a family physician (GP) rather than an otorhinolaryngologist before receiving an HNC diagnosis, with a reported rate of 86% [13]. Unlike Japan, there is a background in which people need to see a GP first, but I think the number is large. However, it was reported that GPs are more likely to unrecognize HNC because they may lack knowledge and experience in it [8]. Furthermore, there are reports that seeing a GP as the initial clinic can lead to treatment delay [5]. Therefore, we believe that it is necessary to encourage patients to consult an otorhinolaryngologist by increasing the awareness of the general public to symptoms suggestive of HNC. 

Notably, the percentage of patients selecting an ENT for the treatment of a neck mass (12.4%) was significantly lower than those choosing an ENT for other symptoms (ranging from 52.6% to 59.0%). This discrepancy is quite significant. Patients tended to opt for internal medicine and dermatology as their medical departments. These findings suggest that the unique nature of a neck mass as a symptom of HNC, and the importance of having it thoroughly assessed by an otorhinolaryngologist, may not be as well understood as other symptoms. It is true that GPs are less likely to encounter HNC patients during their careers [14,15]. However, we believe that it is necessary to make it known to internal medicine clinics, which play the role of GPs, that some cases of HNC who visit a hospital have a chief complaint of neck mass. In addition, we used the term neck mass to collectively refer to thyroid tumors, salivary gland tumors, and cervical lymph node metastases. As a result, a higher proportion of respondents answered that they would consult a dermatologist for a neck mass than for other symptoms. This may be because some respondents did not have the concept of a neck mass and interpreted it as a skin disease on the neck. Furthermore, among the cases in which no answers were obtained, the most common cause was neck mass (11 cases). This may be due to the fact that the patient was unable to select the medical department to be examined. Thus, we believe that there is little recognition that neck masses are diseases in the field of otorhinolaryngology.

A study on the timing of medical visits revealed that patients tended to ignore neck masses more than other HNC-related symptoms. It was reported that patients tend not to seek medical attention for non-painful symptoms [16]. Based on the results, we believe that painless hoarseness and neck mass are symptoms that are easier to ignore than sore throat and pain when swallowing. However, in patients with HNC, the most common symptom is cervical lymphadenopathy, with a reported frequency of 25%-38.2% [14,17]. Therefore, we believe that it is necessary to educate the general public that a neck mass is one of the important symptoms suggestive of HNC and that it is not a symptom that should be ignored. Additionally, for symptoms other than neck masses, many patients said that they would seek medical attention within a week of noticing symptoms. However, in reality, it was reported that the period from when patients with HNC become aware of symptoms until they seek medical attention is two to five months [18-20]. Even if many patients notice some symptoms, it is expected that they will not seek medical attention if these only have a minor impact on their daily lives. We believe that it is important for the general public to understand that not leaving symptoms suggestive of HNC untreated will lead to early detection of HNC and will be the first step in treatment to minimize functional impairment. This is not limited to neck masses.

Limitations

This study targeted patients who did not suffer from HNC. Therefore, differences are expected from the actual movements of patients with HNC. Specifically, since patients with HNC are expected to collect information before consulting a doctor, we believe that in clinical practice, the proportion of patients who visit an otorhinolaryngologist may be a little higher. In addition, regarding the period of time from noticing symptoms to seeing a doctor, there were some answers other than the suggested option, “I will consider the timing of seeing a doctor depending on the severity of the symptoms.” These are considered to be the limitations of a questionnaire survey for symptoms that have not yet happened.

Furthermore, we attempted to avoid bias as much as possible. For example, we excluded patients who visited the hospital for treatment of diseases related to the survey questions. Moreover, we simplified the questionnaire such that even older individuals could respond to it easily. However, we cannot rule out the possibility that bias might have been introduced by not allowing for more open answers or more options.

## Conclusions

The initiation of treatment for patients with head and neck cancer (HNC) is delayed due to various factors in clinical practice. One reason is that patients with HNC do not visit an ENT doctor. This study was conducted to determine the departments that patients with potential HNC symptoms tend to select. Consequently, it was difficult to say that any of the symptoms were sufficiently well-known. Particularly, the awareness about the neck masses was low. The lack of understanding by the general public regarding the relationship between neck masses and HNC is a problem for prompt initiation of treatment.
